# Attitudes Towards Sport in Early Adolescence: A Scale Adaptation Study for Sustainable Good Health and Well-Being

**DOI:** 10.3390/healthcare14070842

**Published:** 2026-03-25

**Authors:** Halil Evren Senturk, Gulsum Tanir, Ulkum Erdogan Yuce, Adem Karatut, Ecesu Karakaş

**Affiliations:** 1 Faculty of Sport Sciences, Mugla Sitki Kocman University, Mugla 48000, Türkiye; ecesukarakas@mu.edu.tr; 2 Institute of Health Sciences, Mugla Sitki Kocman University, Mugla 48000, Türkiye; gulsumtanir@posta.mu.edu.tr; 3 Ministry of National Education, Mugla 48700, Türkiye; ulkumyuce48@gmail.com; 4 Ministry of National Education, Kocaeli 41050, Türkiye; ademkaratut4641@gmail.com

**Keywords:** health-lifestyle behaviors, preventive healthcare, early adolescence, sports attitude, physical literacy, physical activity, scale adaptation, psychometrics, good health, well-being

## Abstract

**Highlights:**

**What are the main findings?**

- The Attitude Towards Sport Scale (ATSS) is a highly valid and reliable measurement tool for early adolescents, successfully preserving its original three-factor structure (Interest, Lifestyle, and Participation).
- The scale scores are strong predictors of actual daily physical activity durations, perceived physical literacy levels, and active athletic status (licensed vs. non-licensed) in middle school students.

**What are the implications of the main findings?**

- Physical education teachers, pediatric public health professionals, and pediatric sports psychologists can use this developmentally sensitive tool to accurately monitor youth sports engagement and identify students at risk of sports dropout.
- Early diagnosis and support of internalized sport attitudes provide an evidence-based pathway to foster lifelong physical activity habits, directly contributing to the targets of Sustainable Development Goal 3 (Good Health and Well-Being).

**Abstract:**

Background: The decline in physical activity during the transition to early adolescence poses a significant threat to lifelong health and well-being, directly impacting the targets of Sustainable Development Goal 3 (SDG 3). To design effective preventive interventions, researchers need developmentally appropriate tools to measure the psychological drivers of physical activity. Objectives: This study aimed to adapt the Attitude Towards Sport Scale (ATSS) for middle school students (ages 10–15) and evaluate its psychometric properties. Methods: We used a mixed-methods approach comprising a qualitative cognitive think-aloud phase (n = 27) and a quantitative cross-sectional validation phase (N = 531). Data were analyzed using robust Confirmatory Factor Analysis (CFA). Results: The results supported the structural model, demonstrating that the original three-factor structure fits the early adolescent sample acceptably. The scale demonstrated high composite reliability across all dimensions. Furthermore, the adapted ATSS-EA showed strong criterion-related validity through high correlations with perceived physical literacy and actual physical activity durations. It also successfully differentiated between licensed athletes and non-licensed students. Conclusions: The adapted ATSS-EA provides a developmentally appropriate tool for educators and researchers to monitor sport attitudes and identify students at risk of physical disengagement.

## 1. Introduction

Globally, approximately 81% of adolescents fail to meet the World Health Organization’s physical activity recommendations [1], a concerning trend that noticeably intensifies during the developmental transition to early adolescence. This decline directly threatens progress toward Sustainable Development Goal 3 (Good Health and Well-Being), which explicitly targets the prevention of non-communicable diseases through early lifestyle modifications.

The United Nations’ Sustainable Development Goals (SDGs), particularly SDG 3, emphasize the promotion of good health and well-being across all ages. A primary mechanism for achieving these targets—specifically in preventing non-communicable diseases and fostering mental health—is the establishment of regular physical activity habits. However, epidemiological evidence shows a sharp decline in sports participation and physical activity during the transition from late childhood to early adolescence, driven primarily by increasing academic pressures and rigorous curriculum demands [1,2]. Furthermore, recent epidemiological evidence highlights a concerning trend: as educational attainment and academic demands increase, adolescents’ physical activity levels tend to decrease while their sedentary time significantly rises [3]. Mitigating this decline is essential for instilling lifelong health behaviors. Since physical activity dropout in youth is not merely a physiological issue but deeply rooted in psychological determinants [4,5], accurately assessing the psychological antecedents of youth behavior is critical for designing sustainable health interventions [2,6]. Therefore, understanding and measuring attitudes towards sport, following the attitude, intention, behavior, and health pathway, indirectly aligns with the preventive health goals of SDG 3 [7,8,9]. Specifically, this psychological assessment aligns with SDG Target 3.4—reducing premature mortality from non-communicable diseases—by targeting physical inactivity (a primary risk factor) at its developmental onset. By measuring sport attitudes during early adolescence, before the well-documented developmental decline in physical activity occurs, researchers can identify the specific affective and cognitive barriers that precede sedentary behavior [10,11,12].

Researchers frequently turn to the Theory of Planned Behavior (TPB) and Self-Determination Theory (SDT) to explain the drivers of sports participation. According to TPB, intention is the most direct cause of a behavior, and this intention is heavily shaped by a person’s attitude—encompassing both their cognitive beliefs and emotional responses [13,14]. Meta-analyses confirm that attitude serves as the primary predictor of physical activity intentions among adolescents [15,16,17,18,19,20]. Complementing this, SDT addresses the nature of motivation. It proposes that healthy habits become sustainable once sports are integrated into a student’s personal identity [21]. This shift from casual participation to making sports a lifestyle choice fits well within SDT’s autonomy continuum [16,22]. Because early adolescence (typically the middle school years) is a key period for this psychological shift [23], reliably measuring these attitudes at a young age is essential for educators and public health professionals working to promote long-term well-being. In the context of preventive medicine, an adolescent’s perspective on sports is more than just a school-related metric; it actively drives their broader health behaviors. Accurately assessing these views provides a foundational step in addressing physical inactivity, obesity, and the non-communicable diseases associated with them.

While many surveys measure general physical activity, the Attitude Towards Sport Scale (ATSS) developed by Senturk [24] captures a more detailed picture by dividing the concept into three areas: Interest in Sport, Sport Lifestyle, and Active Participation. This scale has proven highly reliable in Turkiye and is a common choice for graduate-level research. The limitation, however, is that the original ATSS was validated strictly for older adolescents in high school. Because middle schoolers are at a distinctly different stage of cognitive, emotional, and behavioral development, applying the high school version to them risks measurement errors and undermines the tool’s validity. Developmentally, early adolescents (ages 10–15) are in a transitional phase from concrete to formal operational thinking. Unlike late adolescents, they often struggle with highly abstract concepts, complex hypothetical scenarios, and lengthy retrospective recall. Furthermore, their reading comprehension levels, attention spans, and social motivations (e.g., higher peer dependence) differ substantially. Consequently, administering a high school-level instrument to middle schoolers introduces significant cognitive load, leading to potential misinterpretation of items and systematic measurement errors.

Theoretically, the three dimensions of the ATSS-EA map well onto both TPB and SDT frameworks. The ‘Interest’ dimension largely aligns with the affective attitude component of TPB and intrinsic motivation within SDT, reflecting the inherent enjoyment of physical activity. The ‘Participation’ dimension is closely linked to behavioral intentions and perceived behavioral control in TPB, as well as the need for competence in SDT. Finally, the ‘Lifestyle’ dimension represents integrated regulation within SDT, where sports participation is assimilated into the adolescent’s core identity and daily routine. This situation is also related to the concept of physical literacy. The concept of physical literacy, first conceptualized by Whitehead [25], emphasizes not only physical competence but also motivation and confidence in sport.

This study aims to bridge this methodological gap by adapting the ATSS for a middle school population (ages 10–15) and rigorously testing its psychometric properties using cognitive think-aloud protocols and advanced structural equation modeling on a large sample (N = 531). By providing a developmentally sensitive and statistically validated tool, this research contributes a reliable measurement instrument for behavioral scientists designing preventive public health interventions that support SDG 3.

Based on the theoretical background and the aim of the study, the following hypotheses were formulated:

**Hypothesis 1 (H1).**  *The adapted ATSS-EA will demonstrate an acceptable model fit for the original three-factor structure (Interest, Lifestyle, and Participation) among middle school students.*

**Hypothesis 2 (H2).**  *The scale will exhibit strong convergent and discriminant validity (AVE > 0.50, CR > 0.70) along with a high level of internal consistency (McDonald’s ω > 0.80) for early adolescence.*

**Hypothesis 3 (H3).**  *The scale will provide strong evidence of criterion-related validity by demonstrating a positive and highly significant correlation with a structurally similar and accepted equivalent measurement instrument in the literature.*

**Hypothesis 4 (H4).**  *The scale will statistically significantly differentiate attitudes between students who are and are not licensed athletes, within the scope of known-groups validity.*

**Hypothesis 5 (H5).**  *The ATSS-EA will demonstrate strict measurement invariance across gender and grade levels.*

## 2. Materials and Methods

### 2.1. Study Design and Participants

This study employs a methodological research design with a cross-sectional survey approach. It aims to evaluate the psychometric properties—specifically, the structural and criterion validity and internal consistency—of the Attitudes Towards Sport Scale (ATSS), originally developed for late adolescents, within a younger cohort of middle school students. The research employed a multi-phase mixed-methods design, combining qualitative cognitive interviewing with quantitative psychometric validation to adapt the “Attitude Towards Sport Scale” (ATSS) for middle school students. Before contacting schools for this study, an application was made to the Turkish Ministry of National Education’s “research permits” system, and the necessary research permission was obtained. Prior to data collection, ethical approval was obtained from the Health Sciences Institutional Ethics Committee of Mugla Sitki Kocman University (Protocol No: 240153; 19 November 2024/143). Since the participants were minors, written informed consent was secured from both their legal guardians (parents) and school administrations. In addition to parental consent, verbal and written assent was obtained directly from all participating children prior to data collection. Students were explicitly informed that their participation was entirely voluntary, that they could withdraw from the study at any time without any academic or social consequences, and that no financial or academic compensation was offered for their participation.. Data collection took place between January and June 2025.

All collected data were strictly anonymized by assigning numerical codes instead of names. The digital dataset was stored on a password-protected and encrypted local drive accessible only to the primary research team. These data security protocols and all related research procedures were fully reviewed and approved by the Muğla Sıtkı Koçman University Ethics Committee prior to the commencement of the study.

In the quantitative phase, a convenience sampling method was employed to select schools within the researchers’ accessible district (Marmaris, Muğla, Turkiye). Within these schools, a stratified approach was used to ensure balanced representation across grades 5 through 8. Although the main study sample was determined to be 600 students, 577 middle school students were included in the study with the consent of accessible parents, and from this number, 531 health data suitable for inclusion in the analysis were obtained to determine the final sample size (Table S1). Due to ethical guidelines (voluntariness and confidentiality), demographic data could not be collected from students who did not give consent or participate in the study and, therefore, a systematic comparison with the participants could not be made. In this application of the study, a stratified purposive sampling method was used in 15 public schools (7 rural/agricultural, 8 city/touristic) (Table S2) selected from Marmaris where the researchers reside, to ensure representation across all grade levels (10 licensed male, 10 non-licensed male, 10 licensed female, and 10 non-licensed female per school; total 600 students). Furthermore, while students reported their active athletic license status themselves, this information was verified by collaborating physical education teachers to ensure accuracy. Licensed status specifically refers to official interscholastic or federation-level competitive sports (verified via the national registry), excluding informal intramural school activities. With the approval of the school administrations and physical education teachers, students from the fifth, sixth, seventh, and eighth grades were invited to participate. It was specifically emphasized that participation was based solely on interest in the research and that no academic advantage or exemption from classes was offered. Students were informed that a study on “physical activity habits” would be conducted, and consent forms were distributed to those who wished to participate, to be given to their parents. One week later, all forms (N = 577) left at the schools’ guidance and counseling offices were evaluated, and groups of five students were formed according to grade level. All students (N = 600) who received the forms at the selected middle schools were initially eligible to participate in the study; however, cases with undelivered parental consent forms, missing data, or identified as multivariate outliers were subsequently excluded from the analyses.

These groups were invited to a guidance and counseling office on days and times deemed appropriate by the school administrations. During 5 min preliminary interviews, a conversation took place regarding the research, the researchers, and how to contribute to the study. Following this enlightening discussion, silence was observed, and the students completed the scale prepared with the revised items. It was reported that all participants completed the scales without encountering problems such as ambiguity or mis-reading, that no incomplete or incorrectly completed scales were found, and that the average time to complete the scale was 14.8 min for each student. The scale forms were carefully compiled into a dataset, anonymized, and stored on the responsible researcher’s personal computer for confirmatory factor analysis. The demographics of the students (N = 531) who participated in the quantitative part of the study and whose data were included in the confirmatory factor analysis after outliers and normality testing are presented in the table below (Table 1).

### 2.2. Data Collection Instruments

#### 2.2.1. Attitude Towards Sport Scale (ATSS)

The “Attitude Towards Sport Scale” (ATSS), developed by Şentürk [24], was utilized as the primary assessment tool. The adapted scale consists of 25 items across three sub-dimensions: Interest (13 items), Lifestyle (6 items), and Participation (6 items). Items are rated on a 5-point Likert scale (ranging from 1 = Strongly Disagree to 5 = Strongly Agree, with 3 serving as a neutral ‘Undecided’ midpoint). In its original validation study among late adolescents, the scale accounted for 60.6% of the total variance and exhibited a high internal consistency coefficient (Cronbach’s Alpha = 0.97). The adaptation process ensured that the items reflect the psychomotor and social development characteristics of middle school students.

The original ATSS was developed utilizing a robust mixed-methods approach, theoretically grounded in the affective and cognitive components of the Theory of Planned Behavior. Beyond strong internal consistency (α > 0.80), the original validation demonstrated excellent construct validity via CFA (e.g., RMSEA < 0.08, CFI > 0.90) and solid criterion-related validity among older adolescents. Since its publication, the scale has been widely utilized in regional descriptive and correlational studies to monitor youth sports engagement, though its application in longitudinal intervention designs remains an area for future expansion. The original high-school ATSS comprises a total of 25 items distributed across three theoretically distinct factors: Interest (13 items), Lifestyle (6 items), and Participation (6 items). During the current early adolescent adaptation process, the structural and theoretical integrity of the original instrument was strictly maintained; absolutely no items were deleted or entirely removed from the scale. Instead, all 25 original items were retained and underwent careful linguistic and developmental modifications (e.g., replacing abstract terminology with concrete equivalents). A comprehensive cross-walk detailing the original high school items, the adapted early adolescent items, and each linguistic modification is provided (Table S3).

Early adolescents (10–15 years old) are in the transitional stage of developing their emerging abstract cognitive capacities, unlike the fully consolidated abstract reasoning seen in older high school students. To confirm whether the statements in the 25 items were correctly understood by this age group, expert opinions (5 physical education teachers and 5 developmental psychologists) were sought, and the clarity of the items was tested using a “think-aloud” protocol with a small group of 24 participants. Consistent with the modern validity framework described by Messick [26] and Padilla and Benítez [27], validity evidence based on response processes was gathered. A ‘Think-Aloud Protocol’ was implemented following the guidelines of Ericsson and Simon [28] and Willis [29] to detect potential semantic ambiguities specific to the early adolescent developmental stage [30]. In particular, Woolley et al. [30] used the “Cognitive Pretesting” method to understand the cognitive processes children experience while reading and interpreting questionnaire items, and proved how critical this is for “Developmental Validity”. In this context, the attitude statements or sentences that make up the scale items were evaluated and rewritten in a way that did not distort their meanings. The details of this application are explained comprehensively in the qualitative phase section of the study.

#### 2.2.2. Physical Activity Questionnaire for Older Children (PAQ-C)

The Turkish adaptation of the PAQ-C [31], originally designed to assess general moderate-to-vigorous physical activity levels over a 7-day period, was conducted by Sert and Temel [32] for middle school populations. It was hypothesized that students exhibiting more positive attitudes on the ATSS-EA would correspondingly report higher actual physical activity scores on the PAQ-C. The scale consists of 9 items and assesses students’ moderate-to-vigorous physical activity (at school, during recess, after school) over the past 7 days. It is scored on a scale of 1 to 5, with an average score taken. While the ATSS-EA measures attitude toward sports, the PAQ-C measures actual behavior, a complementary construct. For the current sample in this study, the Cronbach’s Alpha internal consistency coefficient was calculated as 0.78.

#### 2.2.3. Perceived Physical Literacy Scale for Middle School Students

To assess the cognitive, motivational, and physical competence aspects of active lifestyle, this scale, recently developed and validated for Turkish middle school students by Akarsu et al. [33], was employed. We hypothesized a strong positive correlation between ATSS-EA dimensions and physical literacy, as both constructs inherently share the underlying dimensions of motivation and sport-related knowledge. The scale consists of 17 items and 4 sub-dimensions. It is a 5-point Likert-type scale. In this study, the Turkish adaptation of the original form was used. The Cronbach’s Alpha coefficient for the current sample was found to be 0.89.

#### 2.2.4. Personal Information Form

The study also utilized a personal information form to gather information on students’ demographic characteristics. This form aimed to ascertain students’ gender, grade level, academic achievement (grade point averages (GPA) out of 100 in Turkiye are available online to students and parents), daily mandatory physical activity time (min) [Question: Approximately how many minutes in total do you spend on physical activities (light-paced walking, cycling, climbing stairs, etc.) that you are required to do in your daily routine (going to school, going to the market, etc.)?], weekly voluntary physical activity time (days × min) [Question: How many days a week and approximately how many hours a day do you dedicate to voluntary intense physical activities (training, exercise, competition, etc. involving running, jumping, skipping rope, etc.)?], and formal sports participation status [Question: Have you ever held a sports license (school sports or sports club)?] by the researchers. In Türkiye, individuals cannot participate in any official sports competition without formal registration; i.e., without a sports license. Official procedures are usually handled by the student/athlete’s coach or teacher, with the consent of their guardian and a medical report.

### 2.3. Qualitative Phase: Cognitive Validity and Think-Aloud Protocol

#### 2.3.1. Participants and Recruitment of Qualitative Phase

A group of 27 students was selected on a voluntary basis from a middle school with a total population of 738 students. No specific exclusion criteria were applied to the school or the students; instead, a stratified purposive sampling method was employed within a selected public school to ensure representation across all grade levels. With the approval of the school administration and physical education teachers, students from the fifth, sixth, seventh, and eighth grades were invited to participate. Prior to the start of the first lesson, a general announcement was made in the schoolyard. Students were informed that a discussion regarding “physical activity habits” would be conducted and those willing to participate were invited to register at the Psychological Counseling and Guidance (PCG) office, while others proceeded to their classrooms. It was strictly emphasized that participation was based solely on interest in the research, with no academic advantages or exemptions from lessons offered.

Following this recruitment process, 27 volunteers were identified with a mean age of 156.3 months (13.03 years, SD = 1.1). These 27 qualitative participants were drawn from the same schools but were explicitly excluded from the subsequent main quantitative sample. The group consisted of 14 males and 13 females, distributed as follows: 7 students from the 5th grade, 6 from the 6th grade, 7 from the 7th grade, and 7 from the 8th grade; 14 licensed and 13 non-licensed by their school (Table S4). Written informed consent was obtained from the parents over a one-week period, specifically covering both participation and audio recording of the sessions. Thematic saturation regarding item comprehension issues was achieved after approximately 20 interviews; however, a total of 27 interviews were completed to ensure an equal and balanced representation across all four middle school grade levels (5th to 8th grades).

The qualitative data were analyzed using Willis’ (2004) [29] cognitive interviewing framework, focusing on four stages: comprehension, retrieval, judgment, and response. Two independent researchers coded the identified ‘error codes.’ Any coding disagreements were resolved through discussion and consensus with a third senior researcher.

#### 2.3.2. The Process of the Think-Aloud Protocol

To identify semantic problems in the items of the original scale developed for high school students, the guidelines suggested by Willis [29] were followed. In accordance with the Think-Aloud Protocol, a valid method that provides direct access to an individual’s cognitive processes [26,28,30,34], students were invited individually to the school’s PCG office for face-to-face sessions. Each session began with a five-minute preliminary interview covering the study’s objectives and the nature of the protocol. This was followed by a warm-up exercise where students practiced verbalizing their internal thought processes. The warm-up exercise involved a neutral cognitive task (e.g., ‘Please count how many windows are in your house and think aloud while counting’) to familiarize students with the process of verbalizing inner thoughts. Once the students demonstrated proficiency in the protocol, they were asked to read the original items (developed for high school students) aloud. During this phase, the researcher remained silent to avoid interference, providing only non-directive prompts such as “Please continue to share what you are thinking” when a student fell silent. Retrospective probing was deliberately chosen over concurrent probing to avoid disrupting the adolescents’ natural cognitive flow. Given the developing working memory capacity of early adolescents, interrupting them after every item could artificially inflate cognitive load and alter their natural response process. Standardized retrospective probes were utilized to assess comprehension and retrieval. Examples of these probes included: ‘Can you explain in your own words what this sentence is asking?’, ‘Were there any specific words in this item that were difficult to understand?’, and ‘How did you decide on the answer you chose?’.

Upon completion of the verbalization phase, a retrospective probing stage was initiated. Abstract concepts that students found difficult to comprehend were meticulously noted to facilitate the subsequent transcription process. The audio recordings were then transcribed verbatim, and error codes (e.g., reading errors, misinterpretations, conceptual confusion) were generated. Each session lasted an average of 38 min.

#### 2.3.3. Results of the Think-Aloud Protocol

The qualitative data were evaluated through face-to-face consultations with an expert panel comprising two physical education teachers and two developmental psychologists. The panel reviewed expressions that students found ambiguous or difficult to understand and revised them into concrete statements appropriate for the developmental level of middle schoolers. Based on the two rounds of cognitive interviews, item revisions were categorized into two main areas to ensure developmental appropriateness for early adolescents: (1) Clarifying abstract concepts: 6 items containing abstract sporting terms were replaced with more concrete, daily life physical activities. (2) Wording substitutions: 4 items underwent vocabulary simplification (e.g., the word “social status” was replaced with “being successful”). For instance, replacing the adult-centric term ‘social status’ with the developmentally appropriate equivalent ‘being successful’ was carefully evaluated. Qualitative feedback confirmed that for early adolescents, social recognition among peers is primarily conceptualized through observable success rather than abstract socioeconomic status, thereby preserving the original theoretical construct while maximizing developmental comprehension. Consequently, the original 25-item scale was reconstructed using language easily comprehensible to the target population.

Direct feedback from the early adolescents was instrumental in reshaping the items. For instance, regarding an item containing the adult-oriented term ‘physiological responses’, a 6th-grade student remarked, ‘I don’t know what physiological means; does it mean sweating or my heart beating fast?’ Consequently, this abstract term was revised to concrete developmental equivalents (e.g., ‘breathing and getting tired’). Similar concrete adjustments are fully detailed in supplementary tables.

To ensure cognitive validity and capture a fresh perspective while minimizing recall bias, an iterative process was implemented. Fourteen days after the initial sessions, a second round of individual think-aloud protocols was conducted in the PCG office with the same students. In this second application, no negative feedback was received, and the clarity of each revised item was confirmed through both researcher notes and audio transcriptions. The findings from this second iteration were shared with the expert panel via email, and the protocol was successfully concluded. The final refined items were then formatted into the scale, ready for administration to the larger sample group for further psychometric testing. Because the primary objective of this qualitative phase was strictly linguistic evaluation (item comprehension) rather than measuring a longitudinal change in their actual sport attitudes, traditional ‘testing effects’ (i.e., memory of previous responses) did not confound the results. In fact, having the same participants review the revised items 14 days later provided a methodological advantage, allowing them to confirm that the specific linguistic barriers they previously identified had been successfully resolved.

One of the strongest aspects that distinguishes this research from similar studies in the literature is that the quantitative analyses are supported by evidence of cognitive validity. “Think-Aloud” protocols ensured that the scale items were stripped of abstract statements at the high school level and adapted to the concrete operational stage of early adolescence. Participants’ verbal interpretation of the items ensured that the items were not only statistically but also semantically valid. This meets the principle of “validity based on response processes,” which is often neglected in scale adaptation studies but is critical for a Behavioral Sciences perspective [27,29,35,36,37].

### 2.4. Data Analysis

In the quantitative phase, the main study sample consisted of 577 middle school students selected via stratified purposive sampling from 15 public middle schools. Prior to CFA, the dataset was rigorously screened. Statistical analyses to prevent model bias were performed using IBM SPSS Statistics 26.0. Univariate outliers were removed based on standardized Z-scores (±3.29), and multivariate outliers were excluded using Mahalanobis distance (χ^2^ > 52.62, *p* < 0.001), resulting in a final sample of 531 students [38,39,40]. As a result of these rigorous screening procedures, 46 problematic observations that could potentially distort the factor structure were removed. A subsequent missing data analysis confirmed that these 46 excluded cases were distributed randomly across the 15 schools, grade levels, and genders, ensuring no systematic demographic bias was introduced by their removal. The remaining refined dataset of 531 participants fully satisfied the foundational assumptions required for Confirmatory Factor Analysis.

Prior to conducting the CFA, univariate normality was assessed. The skewness and kurtosis values for all items were found to be within the acceptable range of −1.5 to +1.5 (or −2.0 to +2.0 depending on your exact data). Given these acceptable normality parameters, treating the 5-point Likert scale data as continuous and employing the Maximum Likelihood parameter estimates with standard errors and a mean-adjusted chi-square test statistic (MLR) was deemed statistically appropriate and robust against potential minor deviations from multivariate normality.

Construct validity was tested via Confirmatory Factor Analysis (CFA) using JASP software (Version 0.95.4), which is powered by the lavaan R package. The Robust Maximum Likelihood (MLR) estimator was utilized to estimate the model parameters. The Maximum Likelihood with Robust standard errors (MLR) estimator was specifically employed, as it is robust to the slight non-normality often inherent in ordinal 5-point Likert scale data. Model fit was evaluated using multiple indices based on the recommendations of Hair et al. [41] and Kline [39]: the ratio of Chi-square to degrees of freedom (χ^2^/df < 5.0), the Comparative Fit Index (CFI ≥ 0.90), the Tucker–Lewis Index (TLI ≥ 0.90), and the Root Mean Square Error of Approximation (RMSEA < 0.08) [42]. Reliability was rigorously assessed by computing Composite Reliability (CR) and Average Variance Extracted (AVE) values [43].

To establish construct validity, known-groups validity was examined by comparing the ATSS-EA scores of licensed athletes and unlicensed students using independent samples *t*-tests. This analytical step was grounded in the well-established hypothesis that licensed adolescents or those engaged in organized sports exhibit significantly more positive attitudes towards sports and physical activity compared to their non-licensed peers [44,45,46,47,48,49]. Additionally, criterion-related validity was established by examining the Pearson correlation coefficients between the ATSS-EA subscale scores and the students’ self-reported ‘physical activity durations’, hypothesizing a positive association between attitudinal levels and actual physical engagement. All preliminary and comparative analyses were performed using IBM SPSS Statistics 26.0.

Although multiple statistical tests and correlational analyses were conducted, traditional alpha adjustments (e.g., Bonferroni correction) were not applied. This decision is theoretically justified as this is a confirmatory, hypothesis-driven psychometric validation study where variables are expected a priori to be highly interrelated. In such contexts, applying overly conservative corrections like Bonferroni would unacceptably inflate Type II error rates and obscure true theoretical associations [50,51].

To establish the criterion-related validity (concurrent validity) of the adapted Attitudes Towards Sports Scale (ATSS-EA), two robust and conceptually aligned measurement tools were administered alongside the core instrument. Based on behavioral theories suggesting that attitudinal precursors directly influence actual physical behavior and self-efficacy, we utilized the scales, “Physical Activity Questionnaire for Older Children” (PAQ-C) and “Perceived Physical Literacy Scale for Secondary School Students” (PPLS). Pearson product-moment correlation coefficients (r) were calculated to evaluate the strength and direction of these hypothesized associations. Significant positive correlations between the ATSS-EA and both external criteria were considered strong empirical evidence of the scale’s criterion-related validity.

## 3. Results

### 3.1. Measurement Model Fit and Structural Validity

Construct validity of the adapted 25-item scale was evaluated using Confirmatory Factor Analysis (CFA) with a robust maximum likelihood estimator. The hypothesized three-factor measurement model (Interest, Lifestyle, and Participation) demonstrated an excellent fit to the empirical data of the middle school sample.

While the Chi-square statistic was significant (χ^2^ = 693.582, df = 272, *p* < 0.001)—a common statistical artifact in studies with large sample sizes—the relative Chi-square ratio (χ^2^/df = 2.55) indicated a strong fit, falling well below the conservative threshold of 3.0. Furthermore, all absolute and incremental fit indices strongly supported the structural adequacy of the model: CFI = 0.968, TLI = 0.964, SRMR = 0.035, and RMSEA = 0.054 (90% CI: 0.049, 0.059) [42,52]. These robust indices definitively confirm the structural validity of the adapted multi-dimensional scale for early adolescents. Consequently, these robust fit indices provided strong empirical support for Hypothesis 1 (H1), definitively confirming the acceptable model fit of the original three-factor structure among middle school students.

To verify multidimensionality, a single-factor model was tested, which yielded poor fit indices (e.g., CFI = 0.815, RMSEA = 0.129). Thus, the superiority of the three-factor structure was confirmed. Furthermore, no post hoc modification indices (e.g., allowing error terms to correlate) were employed. This strict methodological decision was made to preserve the original scale’s a priori theoretical structure and to avoid the severe risk of capitalizing on chance (i.e., sample-specific overfitting), ensuring that the confirmed model represents true theoretical construct validity rather than mathematical manipulation. To further examine discriminant validity, the shared variances (r^2^ ranging from 0.52 to 0.64) were evaluated in the context of the Average Variance Extracted (AVE) values. While the shared variance between the most highly correlated dimensions approaches their respective AVEs, the unequivocally poor fit of the alternative unidimensional model provides robust statistical confirmation that these factors, while highly related, are empirically distinct and multidimensional constructs.

### 3.2. Convergent Validity and Reliability

**Figure 1:**
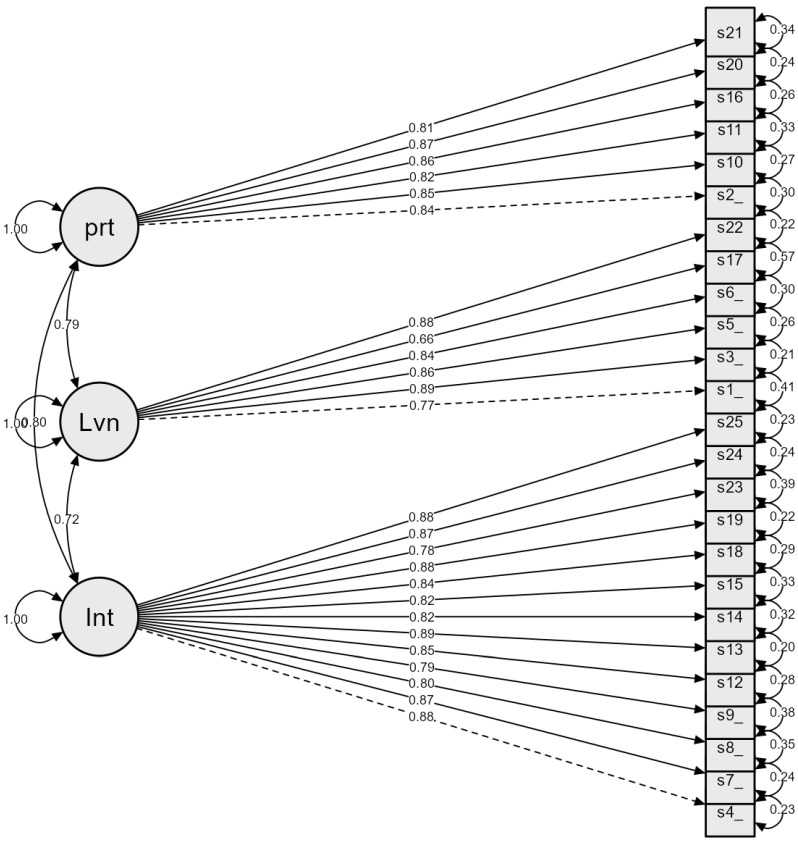
Confirmatory Factor Analysis Path diagram of the ATSS-EA.

As detailed in the parameter estimates, all items loaded strongly and significantly onto their respective latent constructs (Table 2). Standardized factor loadings (λ) ranged from 0.66 to 0.89 (all *p* < 0.001), well above the recommended threshold of 0.60, indicating that the items effectively capture the underlying dimensions without significant redundancy (Figure 1).

Convergent validity was further established through Average Variance Extracted (AVE) and Composite Reliability (CR) metrics. The AVE values for Interest (0.717), Lifestyle (0.679), and Participation (0.709) substantially exceeded the recommended 0.50 threshold. Furthermore, while not directly computed by the software, the robust metrics indicate excellent internal consistency and construct reliability, demonstrating that the latent factors explain a significant portion of the variance in their indicators [43].

Discriminant validity was confirmed by examining the inter-factor correlations. The correlations among the three latent constructs ranged from 0.72 to 0.80. Since no correlation coefficient exceeded the severe multicollinearity threshold of 0.85 [40], it was concluded that the sub-dimensions measure distinct, albeit related, aspects of sports attitudes.

Finally, the internal consistency of the scale was robust. Both Cronbach’s alpha (α) and McDonald’s omega (ω) coefficients yielded values ≥ 0.92 across all three sub-dimensions (Interest: ω = 0.971, α = 0.970; Lifestyle: ω = 0.927, α = 0.923; Participation: ω = 0.936, α = 0.935), providing strong evidence for the reliability of the adapted instrument for early adolescents. Collectively, the metrics of adequate average variance extracted (AVE > 0.50), composite reliability (CR > 0.92), and excellent internal consistency fully supported Hypothesis 2 (H2).

To ensure the scale’s transferability and practical utility across different student subgroups, measurement invariance (configural and metric) was tested across gender and grade levels. The multi-group CFA results demonstrated that the three-factor structure and factor loadings remained invariant across both sex and grade groups [53]. A ΔCFI ≤ −0.010 and a ΔRMSEA ≤ 0.015 indicate that measurement invariance is supported [53]. Detailed invariance statistics are provided in the Supplementary Materials (Table S5).

To provide further evidence for construct validity, the known-groups method was employed (Table 3). As expected, independent samples *t*-test results revealed that licensed student-athletes scored statistically significantly higher across all sub-dimensions of the ATSS-EA compared to their non-licensed peers (*p* < 0.001). This significant differentiation underscores the scale’s robust known-groups validity, demonstrating its capacity to reflect accurately individuals’ real-life behavioral statuses (e.g., participating in organized sports, holding an athletic license, attending regular training) [54,55,56]. The scale’s strong discriminative capacity suggests it is a highly practical screening instrument for physical education teachers and sports psychologists in talent identification and attitude monitoring. These robust findings strongly support Hypothesis 4 (H4), verifying that the adapted scale statistically significantly differentiates attitudes between licensed athletes and non-licensed students.

To rigorously establish known-groups validity and address potential demographic confounders, an Analysis of Covariance (ANCOVA) was conducted. The ATSS-EA total score was entered as the dependent variable, while athletic license status (licensed vs. non-licensed) served as the fixed factor. Gender and grade level were entered as covariates to control for their potential confounding effects. The ANCOVA results demonstrated that after adjusting for grade and gender differences, license status remained a highly significant and robust predictor of sports attitudes (Table 4). This robust finding confirms that active engagement in formal sports significantly differentiates early adolescents’ attitudes, independent of their age or gender. We also found no differences in ATSS-EA between the four grade level (F(3, 527) = 2.83, *p* = 0.038) using ANOVA and *t*-test (t = 0.322, *p* = 0.747) (Tables S6 and S7). Thus, Hypothesis 5 (H5), which posits that ATSS-EA shows absolute measurement invariance across gender and class levels, has been confirmed. The lack of significant attitudinal differences across grades 5 through 8 is theoretically noteworthy. Rather than being surprising, this indicates a period of relative psychological stability in sport attitudes during early adolescence, occurring just before the steeper, well-documented declines in physical activity participation that typically manifest in later high school years.

Criterion-related validity was further established through Pearson correlations between the ATSS-EA sub-dimension scores and two behavioral criteria: daily mandatory physical activity time (DMPAT) and weekly voluntary physical activity time (WVPAT) (Table 5). Interestingly, while attitude factors showed weak albeit significant associations with mandatory physical activities (DMPAT r values ranging from 0.095 to 0.121), they were moderately to strongly correlated with voluntary physical activity durations (WVPAT r values ranging from 0.422 to 0.523, *p* < 0.001). This theoretically sound distinction indicates that internalized sport attitudes are predominantly reflected in voluntary athletic engagement rather than compulsory school activities. Consistent with Eime et al. [44], these sustained voluntary behaviors act as a psychological and social shield, reinforcing the protective role of sports participation during early adolescence [57,58].

To establish criterion-related validity (specifically, concurrent validity) for the adapted ATSS-EA, Pearson product-moment correlation coefficients were calculated (Table 6). This analysis examined the bivariate relationships between the ATSS-EA dimensions (Interest, Lifestyle, and Participation) and two established external health and behavioral criteria: the Perceived Physical Literacy Scale (PPLS; comprising Motivation, Information, Trust, and Physical dimensions) and a physical activity measure (PAQ-C: Physical Activity Questionnaire for Children). Most notably, the overall ATSS-EA score demonstrated a strong correlation with overall physical literacy (r = 0.923) and physical activity levels (r = 0.845). When examining the sub-dimensions, a strong theoretical alignment is observed. The Interest dimension, which primarily captures the cognitive and affective components of sports attitude, exhibited its highest correlations with the cognitive/psychological facets of physical literacy, specifically motivation (r = 0.841), information (r = 0.823), and trust (r = 0.830).

Conversely, the behavioral dimensions of ATSS-EA (Lifestyle and Participation) showed good agreement with behavioral outcomes. The Lifestyle dimension showed its strongest internal physical literacy correlation with the physical dimension (r = 0.739). Furthermore, active physical engagement, measured by the PAQ-C, correlated highly with both the Lifestyle (r = 0.755) and Participation (r = 0.757) dimensions. This nuanced correlational matrix confirms that the adapted ATSS-EA does not merely assess a superficial inclination towards sport; rather, it effectively captures a deep-seated behavioral intent that manifests directly in early adolescents’ physical literacy and actual daily physical activity habits. As detailed in Table 5, the analyses yielded positive, statistically significant, and strong correlations across all theoretical dimensions (*p* < 0.001), thereby providing robust empirical support for Hypothesis 3 (H3).

## 4. Discussion

### 4.1. Theoretical Implications and Synthesis

To our knowledge, this is the first study to systematically adapt a multidimensional sport attitude scale specifically for early adolescents, uniquely combining rigorous cognitive pretesting to ensure developmental linguistic appropriateness with robust psychometric validation.

Early adolescence is one of the critical developmental thresholds for establishing physical activity habits, though it is acknowledged that sport adoption can also occur in later life stages [59]. In line with the good health and well-being, protecting public health depends on accurately diagnosing the psychological foundations of this abandonment [60,61,62,63,64,65,66]. The findings of this study demonstrate that the Attitude Towards Sport Scale (ATSS-EA) is a highly valid, reliable, and developmentally appropriate tool for measuring internalized attitudes towards sport in individuals during early adolescence.

One of the study’s findings that contributes to the literature is the high correlation between attitude dimensions and perceived physical literacy and actual physical activity levels. Our findings reveal a very strong match between the Interest dimension of ATSS-EA and the cognitive (knowledge) and emotional (motivation, confidence) dimensions of physical literacy, demonstrating an acceptable agreement with theoretical expectations. Furthermore, the direct prediction of physical activity duration by the Lifestyle and Participation dimensions proves the behavioral validity of the instrument. As emphasized in current healthcare publications [67,68,69,70,71,72,73,74,75], internalizing physical literacy and a positive attitude towards sport at an early age is a vital preventive medicine strategy against global health crises such as obesity and sedentary lifestyles. Because the adapted ATSS-EA scores exhibited a strong correlation with actual physical activity durations (PAQ-C), the scale effectively predicts a primary healthcare outcome. Therefore, it transcends being a simple pedagogical tool and emerges as a vital screening instrument for preventive healthcare, enabling professionals to monitor and promote health-lifestyle behaviors during the critical transition of early adolescence.

Our findings regarding construct validity showed that the original three-factor construct (Interest, Sport Lifestyle, and Participation), designed for the high school population, was preserved with excellent fit indices in the middle school sample. High factor loadings and Composite Reliability (CR) values confirm the robust construct validity and internal reliability of the instrument. Although the cognitive capacities of early adolescents are based on more concrete operations compared to late adolescents, the multidimensional nature of attitudes toward sport remains unchanged. This confirms that the “strong link between intention and behavior” predicted by the Theory of Planned Behavior [13] operates with the same structural integrity in early childhood as well. The precise preservation of these three dimensions across developmental stages suggests that the underlying cognitive architecture of sport attitudes is already firmly established by early adolescence. This strongly supports the core assumption of the Theory of Planned Behavior (TPB) that attitudes operate as stable, belief-based constructs guiding behavioral intentions.

Theoretically, the strong inter-factor correlations (e.g., r = 0.80) observed in this study suggest that the dimensions of Interest, Lifestyle, and Participation are closely intertwined within early adolescents’ psychological experience. Rather than functioning as completely orthogonal domains, they operate as highly interdependent facets of a broader, higher-order ‘general sport attitude.’ This theoretical structure empirically justifies the computation and practical use of an overall ATSS-EA Total Score by educators and researchers, as it accurately reflects the holistic nature of youth sports engagement.

An interesting finding of this study is that sport attitudes significantly predicted voluntary physical activity (WVPAT) but showed very weak, non-significant associations with mandatory physical activity (DMPAT). This theoretical divergence is expected: mandatory activities (e.g., compulsory physical education classes or active commuting to school) are largely driven by external obligations, school curricula, or environmental necessities, leaving little room for intrinsic attitudes to influence behavior. Conversely, voluntary sports participation requires internal motivation and positive attitudes, which the ATSS-EA successfully captures.

While the ATSS-EA total score exhibited exceptionally high correlations with perceived physical literacy (r = 0.923) and self-reported physical activity via PAQ-C (r = 0.845), these findings must be interpreted with academic caution. Rather than solely reflecting a definitive prediction of actual physical behavior, effects of this magnitude likely indicate a substantial conceptual overlap among these closely related constructs. Furthermore, the reliance on parallel self-report measurement formats may have contributed to these strong associations. Additionally, self-reported physical activity durations are inherently subject to recall bias. Regarding discriminant validity, while ATSS-EA and PPLS are theoretically distinct constructs (attitude vs. perceived competence), the exceptionally high shared variance observed in this study is likely heavily inflated by common method bias. Because both scales were administered concurrently using parallel self-report formats, this shared method variance artificially amplifies the observed correlations. Future validation studies should incorporate objective physical activity metrics to disentangle this conceptual overlap.

While the affective and motivational components within the PPLS (e.g., valuing physical activity) naturally overlap with the ATSS-EA constructs, they remain theoretically distinct. The ATSS-EA measures the direct psychological evaluation and behavioral intention towards sport (attitude), whereas the PPLS captures a broader self-appraisal of physical competence, knowledge, and understanding (literacy). Nonetheless, as noted in the limitations, the extremely high correlation (r = 0.923) suggests that early adolescents may not fully differentiate between ‘liking sports’ and ‘being physically literate’ when responding to concurrent Likert-type scales. Rather than merely demonstrating concurrent validity, this strong correlation (e.g., between the ATSS-EA Interest dimension and physical literacy) carries profound theoretical weight. It suggests that pedagogical interventions targeting intrinsic enjoyment may simultaneously enhance both positive sport attitudes and perceived physical competence, thereby offering a highly efficient, dual-target mechanism for school-based programs.

Our known-groups validity results revealed that students who are licensed athletes exhibit a massive difference (large effect size) compared to their unlicensed peers in all attitude sub-dimensions. While this is expected, what is particularly noteworthy is that licensed athletes show the greatest difference in the Participation and Lifestyle dimensions. When considered within the context of Deci and Ryan’s [22] Self-Determination Theory, it is seen that licensed athletes internalize sport not merely as an external obligation (a school subject) but as a part of their identity (autonomous motivation). Indeed, recent studies [61,62,63] argue that the fundamental condition for the sustainability of the physical education and sports ecosystem is to ensure that children adopt sport as a way of life (Lifestyle) rather than imposing it on them [66,76,77,78,79,80,81,82,83]. Our adapted ATSS-EA provides a valuable tool to assess this level of ‘internalization’ at an early age.

According to Piaget’s [84] (1972) theory of cognitive development, early adolescents predominantly rely on concrete operational thinking. While this cognitive stage necessitates simpler, non-hypothetical item wording to prevent comprehension errors, our findings suggest that the underlying psychological architecture—the multidimensional three-factor structure of sport attitudes—remains consistent across developmental stages.

### 4.2. Practical Applications for Educators

At the micro-level, physical education teachers can utilize ATSS-EA scores as an early-warning diagnostic tool to identify specific students experiencing declining interest or amotivation. This allows for the timely tailoring of PE curricula to individual developmental needs and provides a standardized metric to evaluate the effectiveness of newly implemented sports programs.

In practice, the multidimensional nature of the ATSS-EA allows for highly targeted public health and educational interventions. For concrete example, if a school district aggregates ATSS-EA scores and finds that students exhibit high ‘Interest’ but low ‘Participation,’ educators and policymakers can deduce that structural, financial, or environmental barriers—rather than a lack of intrinsic motivation—are hindering physical activity. Consequently, intervention resources can be strategically diverted away from redundant motivational campaigns and toward providing accessible, low-cost sports infrastructure or after-school intramural programs.

For professionals on the ground—such as physical education teachers, pediatric psychologists, and public health policymakers—this multidimensional tool functions as an effective screening measure. It allows them to track youth engagement in sports and to quickly spot students who are at risk of dropping out before they adopt sedentary habits. With these insights, educators can design better, more specific interventions. In conclusion, health professionals and educators can use measuring and promoting positive attitudes towards sports during middle school years as a key screening step toward achieving the lifelong well-being goals set out by the United Nations Sustainable Development Goal 3 (SDG 3). Ultimately, utilizing such developmentally sensitive tools is a fundamental step in transitioning youth from compulsory physical activities toward autonomous, sustainable, and lifelong athletic lifestyles.

In conclusion, the ATSS adapted for early adolescents is not only an “attitude screening tool” for physical education teachers or sports psychologists, but also a strategic early warning system for public health professionals and education policymakers. Using this multidimensional scale to identify children’s tendencies to drop out of sports rather than focusing on talent selection, and to develop preventative pedagogical interventions, will directly serve the SDG 3 vision of “healthy generations”.

### 4.3. Limitations and Future Directions

While this study robustly validates the ATSS-EA for early adolescents, several limitations should be acknowledged. First, the sample was drawn exclusively from 15 schools 87 rural, 8 urban) in Marmaris, Mugla, Türkiye. This limitation is the geographic and socioeconomic specificity of the sample. Data were exclusively collected from public schools in Marmaris, a coastal district characterized by a tourism-driven economy and potentially better access to recreational sports facilities compared to inland or rural regions of Turki-ye. Consequently, while the sample is highly representative of this specific demographic, the findings should be generalized with caution. The current validation primarily applies to early adolescents in similar socio-cultural and economic contexts. Although this provided a sufficiently large sample, the relatively homogeneous sociocultural structure of this distinct region may limit the generalizability of the findings. Future research should aim to replicate this study across diverse geographic regions (e.g., eastern or central Turkiye) and varying socioeconomic strata to establish broader national generalizability. Direct data on family socioeconomic status (SES) and specific access to sports facilities could not be collected. Future research should employ longitudinal designs tracking these attitude trajectories from late childhood through late adolescence. Additionally, the ATSS-EA should be utilized as a primary outcome measure in pre/post intervention studies, and further cross-cultural validation studies are required to determine the necessary linguistic and structural adaptations for international use.

Several limitations in the current study offer clear directions for future work. First, testing the ATSS-EA across different cultural and geographic groups will be essential to see how well these findings generalize. Additionally, because we relied on a cross-sectional design, we cannot confirm direct cause-and-effect links between a student’s attitude, their physical literacy, and how active they actually are. To truly understand why early adolescents stop playing sports as they move into high school, researchers will need longitudinal studies that follow these behavioral shifts over a longer period. Furthermore, because participation was entirely voluntary, the study is susceptible to self-selection bias; students who already possess a baseline interest in sports may have been more inclined to return the consent forms, potentially skewing the sample towards more positive attitudes.

A methodological limitation of the current study pertains to the clustered nature of the data. Because participants were recruited from 15 different middle schools, the dataset inherently possesses a nested structure (i.e., students clustered within schools). In our confirmatory factor analysis and correlational assessments, individuals were treated as independent observations. Although we utilized the MLR estimator—which is robust to non-normality and provides adjusted standard errors—failing to explicitly model this multilevel data structure may still lead to an underestimation of standard errors and an inflation of Type I error rates. Future research validating the ATSS-EA should consider employing multilevel structural equation modeling (MSEM) to appropriately partition and account for the variance at both the individual student and school levels.

Another point to consider is our use of self-reported surveys. As is typical in adolescent research, participants might overestimate their activity levels or simply misremember them due to social desirability or recall bias. Pairing the scale with objective tracking tools, such as accelerometers, would give a much more accurate picture of their real-world physical activity. Furthermore, the cross-sectional design of this study prevents the precise determination of causal trends in terms of known groups validity. It remains theoretically unclear whether pre-existing positive attitudes drive adolescents to obtain athletic licenses, or whether the structured, rewarding environment of licensed sports participation enhances these positive attitudes. Future longitudinal studies are necessary to map these developmental trajectories.

A critical limitation of the current study is the potential for common-method bias. Because all primary variables—including sports attitudes, physical literacy, and physical activity durations—were evaluated concurrently using self-report questionnaires, our dataset is highly susceptible to same-source bias. This shared method variance likely inflated the observed correlations between the ATSS-EA and external criterion variables. Future validation studies should incorporate objective physical activity metrics, such as accelerometry or pedometric, to mitigate this bias and provide a more accurate estimation of how well the scale predicts actual behavior.

Additional limitations include the absence of test–retest reliability data and the inherent risk of social desirability bias in self-reported attitudes. Future studies should establish longitudinal test–retest stability and cross-cultural measurement invariance. Moreover, future research should aim to develop clinically meaningful cutoff scores, examine the scale’s sensitivity to change following pedagogical interventions, and triangulate self-reported data with proxy measures such as parent or teacher evaluations.

The extremely high Composite Reliability (CR) values (e.g., >0.930) and strong factor loadings (R^2^ > 0.30 across most items) suggest potential item redundancy. Future psychometric studies should consider developing and validating a brief/short-form version of the ATSS-EA to reduce administration time and cognitive burden in large-scale epidemiological surveys.

Finally, a note of caution is necessary regarding the English version of the ATSS-EA. We provided this translation mainly to help international readers to understand the conceptual framework. Even though a team of bilingual experts carefully handled the translation and back-translation process, we have not yet tested this English version on a native English-speaking middle school sample. Therefore, before anyone uses the English ATSS-EA for actual data collection, it still needs to undergo a full cross-cultural validation study. We strongly encourage international researchers to take up this task to broaden the scale’s global utility.

## 5. Conclusions

Encouraging young adolescents to maintain active lifestyles remains a major public health challenge. To address the lack of age-appropriate measurement tools, this study adapted and validated the Attitude Towards Sport Scale (ATSS) specifically for middle school students. Our psychometric analyses—covering construct, criterion, and known-groups validity—confirm that the 25-item scale is reliable and developmentally suited for this demographic. It successfully retains its original three-factor structure: Interest, Lifestyle, and Participation. Beyond just recording student opinions, the adapted scale proved capable of predicting actual physical activity levels and perceived physical literacy. This means the ATSS-EA serves as a practical behavioral predictor rather than just a simple attitude survey. Ultimately, the ATSS-EA offers a robust, developmentally tailored tool ready for cross-cultural adaptation and application in future longitudinal and intervention research aimed at sustaining youth physical activity.

## Figures and Tables

**Table 1:** Demographics of Students Participating in the Quantitative Section of the Study.

Demographic	Groups	N	%	M_GPA_	M_DMPAT_ (min)	M_WVPAT_ (min)
Gender	Male	258	48.6	87.29 ± 11.97	37.52 ± 19.68	224.19 ± 148.61
	Female	273	51.4	87.42 ± 11.10	30.90 ± 20.27	144.18 ± 112.79
Grades	5.	135	25.4	86.64 ± 13.35	32.59 ± 19.70	166.67 ± 114.74
	6.	142	26.7	89.37 ± 9.63	32.89 ± 20.32	167.75 ± 143.59
	7.	131	24.7	87.63 ± 10.75	35.42 ± 20.10	209.08 ± 146.069
	8.	123	23.2	85.51 ± 11.91	35.81 ± 20.91	190.98 ± 139.46
Licensed	Yes	268	50.5	89.30 ± 10.40	36.66 ± 19.57	235.52 ± 130.49
	No	263	49.5	85.38 ± 12.27	31.52 ± 20.61	129.58 ± 112.24

Note. DMPAT = daily mandatory physical activity time (min); WVPAT = weekly voluntary physical activity time (days × min); GPA = average grade.

**Table 2:** Psychometric Properties of the Adapted Scale (Factor Loadings, CR, and AVE).

Item No	Factor 1 (Interest) (λ)	Factor 2 (Lifestyle) (λ)	Factor 3 (Participation) (λ)	R^2^
4	0.876			0.767
7	0.871			0.759
8	0.803			0.645
9	0.790			0.624
12	0.847			0.717
13	0.894			0.799
14	0.822			0.675
15	0.820			0.672
18	0.843			0.711
19	0.882			0.778
23	0.783			0.613
24	0.874			0.763
25	0.878			0.771
1		0.766		0.587
3		0.890		0.792
5		0.863		0.744
6		0.839		0.705
17		0.656		0.431
22		0.883		0.780
2			0.839	0.703
10			0.852	0.726
11			0.818	0.669
16			0.859	0.738
20			0.871	0.758
21			0.812	0.659
AVE	0.717	0.679	0.709	
CR	0.970	0.925	0.936	
McDonald’s (ω)	0.971	0.927	0.936	0.973 (Total)
Cronbach (α)	0.970	0.923	0.935	0.974 (Total)

Note. χ^2^(272) = 693.582, *p* < 0.001; χ^2^/df = 2.55; CFI = 0.968; TLI = 0.964; RMSEA = 0.054 (90% CI [0.049, 0.059]); All loadings are standardized and significant at *p* < 0.001. CR = Composite Reliability; AVE = Average Variance Extracted. Estimator = Robust Maximum Likelihood (MLR).

**Table 3:** Construct Validity: Known-Groups Comparison.

Factors	Groups	N	M	SD	t	df	*p*	Cohen’s d
Interest	Licensed	268	55.32	10.99	8.515	529	0.001 *	0.739
	Non-licensed	263	45.54	15.18				
Lifestyle	Licensed	268	24.82	4.25	7.991	529	0.001 *	0.694
	Non-licensed	263	21.24	5.94				
Participation	Licensed	268	20.54	3.77	10.114	529	0.001 *	0.878
	Non-licensed	263	16.61	5.10				
ATSS-EA	Licensed	268	104.98	17.74	9.668	529	0.001 *	0.839
	Non-licensed	263	86.91	24.82				

* *p* < 0.001.

**Table 4:** Analysis of Covariance (ANCOVA) for ATSS-EA Scores by License Status, Controlling for Grade and Gender.

Source	Sum of Squares	df	Mean Square	F	*p*	η^2^_p_
**Covariates**						
Grade	3522.774	3	1174.258	2.700	0.045	0.015
Gender	12,727.557	1	12,727.557	29.268	<0.001	0.053
Main Effect						
License Status	34,854.511	1	34,854.511	80.149	<0.001	0.132
Error (Residuals)	228,306.493	525	434.870			

Note. η^2^_p_ = Partial eta squared effect size. Dependent Variable: ATSS-EA.

**Table 5:** Criterion-related validity via Pearson correlations with physical activity durations.

PA Durations	ATSS-EA	Factor 1 (Interest)	Factor 2 (Lifestyle)	Factor 3 (Participation)
DMPAT (min)	0.113	0.095	0.121	0.104
	0.009 *	0.029 *	0.005 *	0.017 *
WVPAT (day × min)	0.492	0.435	0.422	0.523
	0.001 **	0.001 **	0.001 **	0.001 **

Note. * *p* < 0.05; ** *p* < 0.001; r = Pearson’s correlation; ATSS: Attitudes Towards Sport Scale; DMPAT: daily mandatory physical activity time; WVPAT: weekly voluntary physical activity time. The markedly stronger correlations with WVPAT compared to the weak associations with DMPAT structurally indicate that the ATSS-EA measures attitudes toward voluntary, intrinsically motivated sports rather than compulsory physical activities. Correlations with WVPAT represent theoretically meaningful, medium-to-large effect sizes, whereas correlations with DMPAT are negligible.

**Table 6:** Criterion-related validity via Pearson correlations [95% CI] with PPLS and PAQ.

Scales/Sub-Scales	ATSS-EA	Factor 1 (Interest)	Factor 2 (Lifestyle)	Factor 3 (Participation)
PPLS	0.923 * [0.91, 0.94]	0.919 * [0.90, 0.93]	0.767 * [0.73, 0.80]	0.752 * [0.71, 0.79]
PPLS (motivation)	0.802 * [0.77, 0.83]	0.841 * [0.81, 0.86]	0.560 * [0.50, 0.62]	0.647 * [0.60, 0.70]
PPLS (information)	0.810 * [0.78, 0.84]	0.823 * [0.79, 0.85]	0.639 * [0.59, 0.69]	0.657 * [0.61, 0.70]
PPLS (trust)	0.796 * [0.76, 0.83]	0.830 * [0.80, 0.85]	0.589 * [0.53, 0.64]	0.619 * [0.56, 0.67]
PPLS (physical)	0.622 * [0.57, 0.67]	0.514 * [0.45, 0.57]	0.739 * [0.70, 0.77]	0.546 * [0.48, 0.60]
PAQ-C	0.845 * [0.82, 0.87]	0.791 * [0.76, 0.82]	0.755 * [0.72, 0.79]	0.757 * [0.72, 0.79]

Note. * *p* < 0.001; ATSS: Attitudes Towards Sport Scale; PPLS: the Perceived Physical Literacy Scale; PAQ-C: Physical Activity Questionnaire for Children.

## Data Availability

The data that support the findings of this study are available from the corresponding author upon reasonable request.

## Supplementary Materials

The following supporting information can be downloaded at: https://www.mdpi.com/article/10.3390/healthcare14070842/s1, Table S1: Demographics of Included (N = 531) and Excluded (N = 46) Students Participating in the Quantitative Section of the Study; Table S2: Schools and Students; Table S3: (a) Examples (top 5*) of the process of adapting original scale items for the middle school level; (b) Revised statements of the process of adapting original scale items for the middle school level; Table S4: The participants of the Think-Aloud Protocol; Table S5: Detailed invariance statistics: ΔCFI and ΔRMSEA values; Table S6: ANOVA—ATSS mean (Grade); Table S7: *t*-test—ATSS mean (Grade).
