# Supplementary material for: Attitudes Towards Sport in Early Adolescence: A Scale Adaptation Study for Sustainable Good Health and Well-Being
Source: Healthcare (Basel). 2026 Mar 25;14(7):842. doi: 10.3390/healthcare14070842 (PMC13073176; doi:10.3390/healthcare14070842)
Supplement: Supplementary file 1 [file healthcare-14-00842-s001.zip › healthcare-4216438-supplementary.pdf]

Supplementary File(s)

Table S1. Demographics of Included (N=531) and Excluded (N=46) Students Participating in the Quantitative Section of the Study

Demographic	Included Groups	N	%	Excluded Groups	N	%
**Gender**	**Male**	258	48.6	**Male**	35	76,1
	**Female**	273	51.4	**Female**	11	23,9
**Grades**	**5.**	135	25.4	**5.**	10	21,7
	**6.**	142	26.7	**6.**	3	6,5
	**7.**	131	24.7	**7.**	12	26,1
	**8.**	123	23.2	**8.**	21	45,7
**Licensed**	**Yes**	268	50.5	**Yes**	15	32,6
	**No**	263	49.5	**No**	31	67,4

Table S2. Schools and Students

Rural Schools	Students	N		City Schools	Students	N	
Orhaniye	**Included**	40		Ahu Hetman	**Included**	40	
	**Excluded**	5			**Excluded**	1	
Selimiye	**Included**	38		Aksaz	**Included**	34	
	**Excluded**	3			**Excluded**	2	
Söğüt	**Included**	47		Evren Pasa	**Included**	41	
	**Excluded**	4			**Excluded**	1	
Turunç	**Included**	36		Sehit Mehemt Cetin	**Included**	38	
	**Excluded**	6			**Excluded**	0	
Bayır	**Included**	39		Bozburun	**Included**	32	
	**Excluded**	1			**Excluded**	2	
Çetibeli	**Included**	43		Icmeler	**Included**	42	
	**Excluded**	6			**Excluded**	3	
Hisarönü	**Included**	38		Beldibi	**Included**	33	
	**Excluded**	5			**Excluded**	4	
				Marmaris	**Included**	36	
					**Excluded**	3	
		**N**	**%**			**N**	**%**
**TOTAL**	**Included**	**281**	**90,4**	**TOTAL**	**Included**	**296**	**94,9**
	**Excluded***	**30**	**9,6**		**Excluded***	**16**	**5,1**

Note. *The reason for the student exclusion was data cleaning performed before the confirmatory factor analysis.

Table S3a. Examples (top 5*) of the process of adapting original scale items for the middle school level

Item no	Original statement (high school)**	Examples of student profile	Feed-back of students**	Error codes	Revised statement (Middle school)**	Outcome of 2nd Protocol
2.	"...a material gain."	Male, 136 m., 5th grade	"What does 'material' mean, teacher?"	Conceptual confusion	“…to earn money…”	Fully Understood
17.	"...I participate."	Female, 149 m., 6th grade	"Does it include playing outside of school?"	Scope ambiguity	“…to do sports…”	Fully Understood
4.	"...physiological benefit..."	Female, 159 m., 7th grade	"What does 'physiological' mean?"	Lack of concept	“…are good for my health”	Fully Understood
13.	"...social status..."	Male, 136 m., 5th grade	"What does 'social status' mean?"	Lack of concept	“Being successful…”	Fully Understood
20.	"... as role models"	Female, 146 m., 5th grade	"...buying their toy."	Conceptual confusion	“…to be like him/her.”	Fully Understood

* The examples presented in the table are randomly selected from the five most frequently occurring similar expressions. ** Some loss of meaning may be noticeable due to the translation.

Table S3b. Revised statements of the process of adapting original scale items for the middle school level

Item no	Original statement (high school)**	Revised statement (Middle school)**
2.	I participate in sports for both spiritual and material gain.	I want to be a good athlete and earn money through sports.
17.	I participate in sports activities even if they are not managed by experts.	I don't need a teacher or coach to do sports.
4.	I believe that sports have physical, physiological, and psychological benefits.	Exercising, playing games, and being active are good for my health.
13.	The social status that comes with sporting success makes me proud.	Being successful in sports, games, and competitions makes me proud.
20.	I look up to the best athletes in my sport as role models.	There is always an athlete I want to be like him/her.
15.	I don't necessarily need someone with me to exercise.	I don't need a friend to exercise with me.
3.	I feel mentally incomplete on days I don't exercise.	I often feel fun when I exercise.
5.	I participate in sports activities voluntarily and without coercion.	I participate in sports, games, or activities that involve movement of my own free will.
23.	I enjoy watching sports that I don't participate in.	I also enjoy watching sports that I can't play or am not good at.
16.	I've always dreamed of pursuing my sport as an elite (national) athlete.	I dream of playing my sport like the most famous athletes.

** Some loss of meaning may be noticeable due to the translation.

Table S4. The participants of the Think-Aloud Protocol

No	ID	License	Grade	Gender	Age (months)
**1**	1L5M	licensed	5	male	136
**2**	2N5F	non-licensed	5	female	141
**3**	3N5F	non-licensed	5	female	139
**4**	4L5M	licensed	5	male	142
**5**	5L5M	licensed	5	male	143
**6**	6N5F	non-licensed	5	female	146
**7**	7L5M	licensed	5	male	137
**8**	8N6F	non-licensed	6	female	149
**9**	9N6M	non-licensed	6	male	151
**10**	10N6M	non-licensed	6	male	148
**11**	11L6F	licensed	6	female	150
**12**	12L6M	licensed	6	male	152
**13**	13N6M	non-licensed	6	male	155
**14**	14L7M	licensed	7	male	161
**15**	15N7F	non-licensed	7	female	159
**16**	16L7M	licensed	7	male	163
**17**	17L7M	licensed	7	male	164
**18**	18L7F	licensed	7	female	158
**19**	19N7F	non-licensed	7	female	161
**20**	20N7F	non-licensed	7	female	160
**21**	21L8M	licensed	8	male	174
**22**	22L8F	licensed	8	female	170
**23**	23N8M	non-licensed	8	male	171
**24**	24L8M	licensed	8	male	173
**25**	25L8F	licensed	8	female	169
**26**	26N8F	non-licensed	8	female	175
**27**	27N8F	non-licensed	8	female	172

Table S5. Detailed invariance statistics: ΔCFI and ΔRMSEA values

Grouping	Model	χ2	df	p	CFI	TLI	RMSEA	SRMR	ΔCFI	ΔRMSEA
**Gender**	Configural	1118.067	544	< .001	0.923	0.915	0.063	0.053	-	-
	Metric	1146.408	566	< .001	0.922	0.918	0.062	0.058	-0.001	-0.001
**Grade**	Configural	1152.091	544	< .001	0.916	0.908	0.065	0.055	-	-
	Metric	1184.225	566	< .001	0.915	0.910	0.064	0.059	-0.001	-0.001

Note. ΔCFI and ΔRMSEA values were calculated by subtracting the Configural model from the Metric model. A ΔCFI ≤ -.010 and a ΔRMSEA ≤ .015 indicate that measurement invariance is supported (Chen, 2007).

Table S6. ANOVA – ATSS mean (Grade)

Grade	N	Mean	SD	df	F	p
5	135	99.73	21.47	3	2.833	0.038
6	142	93.13	24.60			
7	131	98.05	22.90			
8	123	93.17	23.76			

Note. Type III Sum of Squares

Table S7. T-test – ATSS mean (Grade)

Grade	N	Mean	SD	df	t	p
5-6	277	96.34	23.32	529	0.322	0.747
7-8	254	95.69	23.40			
